# Self-reported Memory Problems 8 Months After COVID-19 Infection

**DOI:** 10.1001/jamanetworkopen.2021.18717

**Published:** 2021-07-29

**Authors:** Arne Søraas, Ragnhild Bø, Karl Trygve Kalleberg, Nathalie C. Støer, Merete Ellingjord-Dale, Nils Inge Landrø

**Affiliations:** 1 Department of Microbiology, Oslo University Hospital, Oslo, Norway; 2 Department of Psychology, University of Oslo, Oslo, Norway; 3 Age Labs AS, Oslo, Norway; 4 Cancer Registry of Norway, Oslo, Norway; 5 Department of Psychology, University of Oslo, Oslo, Norway

## Abstract

This cohort study examines self-reported memory problems 8 months after COVID-19 infection.

## Introduction

COVID-19 is an airway disease that also affects the nervous system.^1^ Therefore, neurological and neurocognitive symptoms may be a part of the postacute sequelae of SARS-CoV-2 infection (PASC) syndrome. PASC may be found to affect a high proportion of people who had mild cases of COVID-19, and there is an urgent need for a detailed description of PASC in nonhospitalized patients.^2,3^ This cohort study examines self-reported memory problems 8 months after COVID-19 infection.

## Methods

This cohort study was approved by the Regional Research Ethics Committee according to the Declaration of Helsinki. Eligible participants provided informed consent by signing an online electronic consent form and completing an online baseline questionnaire and follow-up questionnaires. This study used the Strengthening the Reporting of Observational Studies in Epidemiology (STROBE) reporting guideline.

We followed a cohort of 13 001 adults who were invited after (1) having their clinical specimen analyzed for SARS-CoV-2 at 4 large accredited laboratories in Norway or (2) being randomly selected from the Norwegian population (untested). All adults who were tested for COVID between February 1 and April 15, 2020, were invited (eFigure in the Supplement). Nearly all testing in Norway during that time was on symptomatic patients and free of charge.^4^

We collected data on demographics, underlying medical conditions, symptoms, health-related quality of life from the RAND 36-Item Health Survey, memory problems, and known confounders for memory problems (eAppendix in the Supplement). Data from participants who were hospitalized are not reported in this study.

The main outcome was self-reported memory problems 8 months after infection, and the exposure was SARS-CoV-2 status (ie, positive, negative, or untested). To determine whether differences in the outcome between the exposure groups remained after adjusting for confounding, we applied a multiple logistic regression model that included age, gender, and known confounders for memory problems (RAND-36 items for physical health limitation, pain, feeling energetic, and mood). SPSS version 27 (IBM) and R version 4.0.3 (R Project for Statistical Computing) were used for the statistical computations. Statistical tests were 2-tailed, and the significance level was set to *P* < .05. Data analyses were performed on May 10 and May 13, 2021.

## Results

We sent up to 3 electronic invitations to 53 168 invitees, and after exclusions, 13 001 (24%) participants completed the baseline questionnaire and were followed up for 8 months (Table). The mean (SD) age was 47 (14.3) years, and 8642 (66%) were women.

**Table.** Study Population and Results

Characteristic	No. (%)		
	SARS-CoV-2–positive	SARS-CoV-2–negative	Randomly selected untested
**Inclusion of participants**			
Invited	2155	31 013	20 000
Sex			
Female	1056 (49)	21089 (68)	9600 (48)
Male	1099 (51)	9924 (32)	10 400 (52)
Age, mean (SD), y	49.6 (17.4)	45.9 (16.6)	45.7 (16.6)
Consenting to participate	853 (40)	8095 (26)	4393 (22)
Excluded from analysis^a^	59 (2.7)	117 (0.4)	164 (0.8)
Included for analysis	794 (37)	7978 (25.7)	4229 (21.1)
Sex			
Female	439 (55)	5962 (75)	2241 (53)
Male	355 (45)	2016 (25)	1988 (47)
Age mean (SD), y	47.3 (13.9)	44.8 (13.2)	51.3 (15.3)
Days from SARS-CoV-2 test to baseline, mean (SD)	15.4 (9.4)	16.3 (9.2)	NA
**Self-reported symptoms the last 3 wk before completing the baseline questionnaire**			
Fever	520 (66)	2204 (28)	100 (2)
Dypnea	318 (40)	2277 (29)	219 (5)
Cough	557 (70)	4283 (54)	430 (10)
Fatigue	645 (81)	3544 (44)	458 (11)
Changed sense of smell or taste	546 (69)	767 (10)	56 (1)
Asymptomatic	20 (3)	670 (8)	2461 (58)
**8-mo follow-up questionnaire**			
Participated at 8-mo follow-up	651 (82)	5712 (72)	3342 (79)
Sex			
Female	369 (57)	4327 (76)	1768 (53)
Male	282 (43)	1385 (24)	1574 (47)
Age (at baseline), mean (SD)	48.6 (13.6)	46.3 (13.0)	52.9 (14.5)
Days from SARS-CoV-2 test to 8-mo follow-up, mean (SD)	258 (33.9)	256 (31.9)	NA
**Self-reported symptoms the last 3 wk before completing 8-mo follow-up questionnaire**			
Memory problems past 3 wk	72 (11)	245 (4)	80 (2)
Problems concentrating past 3 wk	81 (12)	409 (7)	135 (4)
**Health-related quality of life and confounders for memory problems at the 8-mo follow-up questionnaire**^**b**^			
Physical health has limited work or other activities past 4 wk	130 (20)	1234 (22)	546 (16)
Pain has limited activities mildly or worse past 4 wk	84 (13)	1025 (18)	534 (16)
Not felt energetic some of the time or more past 4 wk	338 (52)	3188 (56)	1331 (40)
Felt downhearted and blue some of the time or more past 4 wk	68 (11)	632 (11)	263 (8)
Worsening in self-reported health past 1 y^c^	267 (41)	1198 (21)	394 (12)

Abbreviations: NA, not applicable; RT-PCR, reverse transcription–polymerase chain reaction.

^a^ The following groups were excluded: randomly selected participants who reported to be tested at baseline, participants who reported to be hospitalized for SARS-CoV-2 and SARS-CoV-2–negative, or participants who were untested at baseline who reported a positive SARS-CoV-2 real-time RT-PCR test result at any time.

^b^ These are items from the RAND-36 questionnaire and were dichotomized as indicated in the Methods for brevity.

^c^ The published mean value in the Norwegian population for this dichotomized variable is 12.3%.

At follow-up, a mean (SD) of 257 (32) days after baseline, 9705 of 13 001 participants (75%) responded, and 72 of 651 of the participants (11%) in the SARS-CoV-2–positive group reported memory problems. In contrast, 254 of 5712 participants (4%) in the SARS-CoV-2–negative group or 80 of 3342 participants (2%) in the untested randomly selected reported memory problems (Figure).

**Figure.**
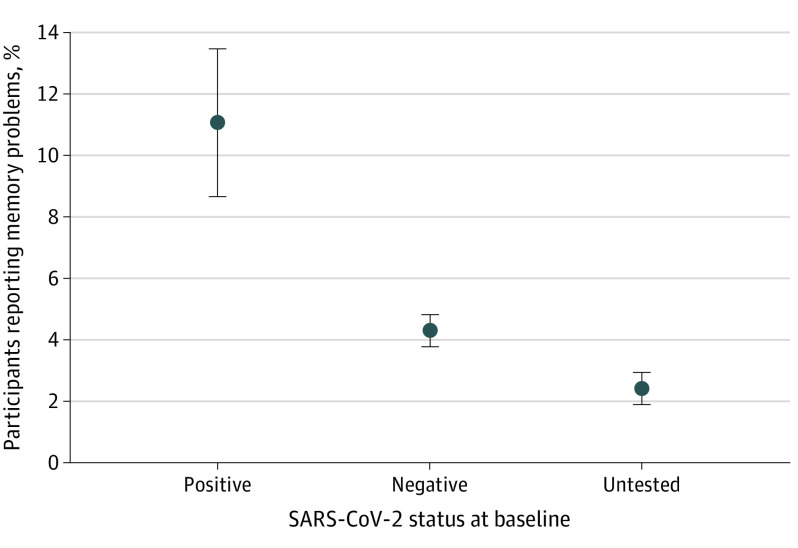
Proportion of Participants Reporting Memory Problems 8 Months After Baseline Error bars indicate SD.

In the multiple logistic regression model, SARS-CoV-2 positivity at baseline was strongly associated with reporting memory problems at 8 months follow-up (odds ratio [OR], 4.66; 95% CI, 3.25-6.66) compared to the untested randomly selected group. At follow-up, 267 of 649 participants (41%) in the SARS-CoV-2–positive group reported a significant worsening of health compared with 1 year prior, and 81 of 651 participants (12%) in the SARS-CoV-2–positive group also reported problems concentrating. Additionally, 59 of 267 participants (82%) in the SARS-CoV-2–positive group who reported memory problems also reported a worsening of health. Feeling depressed, having less energy, or pain were reported relatively equally by the different groups (Table).

## Discussion

We examined the prevalence of self-reported memory problems in a large group of COVID-19 patients who were not hospitalized and had a relatively mild disease. Eight months after the positive SARS-CoV-2 test, the prevalence of memory problems in this group was higher than in the control group with a negative test or in the untested control population.

Most of the SARS-CoV-2–positive participants with memory problems also reported a worsening of their health compared with 1 year prior. Our findings suggest that SARS-CoV-2 may negatively impact memory even 8 months after having a mild case of the disease, and this can be associated with a worsening of health and PASC. The findings are a strong impetus to reconsider the notion that COVID-19 can be a mild disease. It also questions whether the current home-treatment strategies are optimal for the long-term outcome. Our results suggest that memory problems may be a part of PASC, but firmer conclusions should await a longer follow-up period.

This study had limitations. Although we ran multiple logistic regression that adjusted for several likely confounders, there may still have been unmeasured or residual confounding. An additional limitation of the study is that knowledge of COVID-19 status and symptoms at baseline could have led to participation bias or response bias during follow up. The low overall response rate of 24% may limit the generalizability of our findings. A strength of the study is the inclusion of 2 relevant comparison groups.

The lack of objective memory tests limits strong conclusions. However, subjective memory concerns have been shown to reflect objective problems and observable changes in everyday function even when controlling for associated factors, such as depression.^5^ Self-reported memory problems are also a risk factor for later mild cognitive impairment or dementia.^6^ Nevertheless, a more detailed examination of what type of memory problems are specific for PASC, like working memory vs long-term memory, is warranted in future studies.

## Notes

Supplement. **eFigure.** Flow Diagram **eAppendix.** Questionnaire

## References

[1] Kandemirli SG, Dogan L, Sarikaya ZT, . Brain MRI findings in patients in the intensive care unit with COVID-19 infection. Radiology. 2020;297(1):E232-E235. doi:10.1148/radiol.2020201697 32384020 PMC7507997

[2] Norton A, Olliaro P, Sigfrid L, ; ISARIC and GloPID-R Long COVID Forum Working Group. Long COVID: tackling a multifaceted condition requires a multidisciplinary approach. Lancet Infect Dis. 2021;21(5):601-602. doi:10.1016/S1473-3099(21)00043-8 33548193 PMC7906694

[3] Munblit D, Bobkova P, Spiridonova E, . Risk factors for long-term consequences of COVID-19 in hospitalised adults in Moscow using the ISARIC Global follow-up protocol: StopCOVID cohort study. medRxiv. 2021:2021.2002.2017.21251895. doi:10.1101/2021.02.17.21251895

[4] Kjetland EF, Kalleberg KT, Søraas CL, . Risk factors for community transmission of SARS-CoV-2: a cross-sectional study in 116,678 people. medRxiv. 2020:2020.2012.2023.20248514. doi:10.1101/2020.12.23.20248514

[5] Landrø NI, Fors EA, Våpenstad LL, Holthe Ø, Stiles TC, Borchgrevink PC. The extent of neurocognitive dysfunction in a multidisciplinary pain centre population: is there a relation between reported and tested neuropsychological functioning? Pain. 2013;154(7):972-977. doi:10.1016/j.pain.2013.01.013 23473784

[6] Reisberg B, Shulman MB, Torossian C, Leng L, Zhu W. Outcome over seven years of healthy adults with and without subjective cognitive impairment. Alzheimers Dement. 2010;6(1):11-24. doi:10.1016/j.jalz.2009.10.002 20129317 PMC3873197

