# Supplementary material for: Self-reported Memory Problems 8 Months After COVID-19 Infection
Source: JAMA Netw Open. 2021 Jul 29;4(7):e2118717. doi: 10.1001/jamanetworkopen.2021.18717 (PMC8322992; doi:10.1001/jamanetworkopen.2021.18717)
Supplement: Supplement. — eFigure. Flow Diagram eAppendix. Questionnaire [file jamanetwopen-e2118717-s001.pdf]

Supplemental Online Content

Søraas A, Bø R, Kalleberg KT, Støer NC, Ellingjord-Dale M, Landrø NI. Self-reported memory problems 8 months after COVID-19 infection. *JAMA Netw Open*. 2021;4(7):e2118717. doi:10.1001/jamanetworkopen.2021.18717

eFigure. Flow Diagram

eAppendix. Questionnaire

This supplemental material has been provided by the authors to give readers additional information about their work.

© 2021 Søraas A et al. *JAMA Network Open*.

eFigure 1. Flow diagram

Inclusion of participants

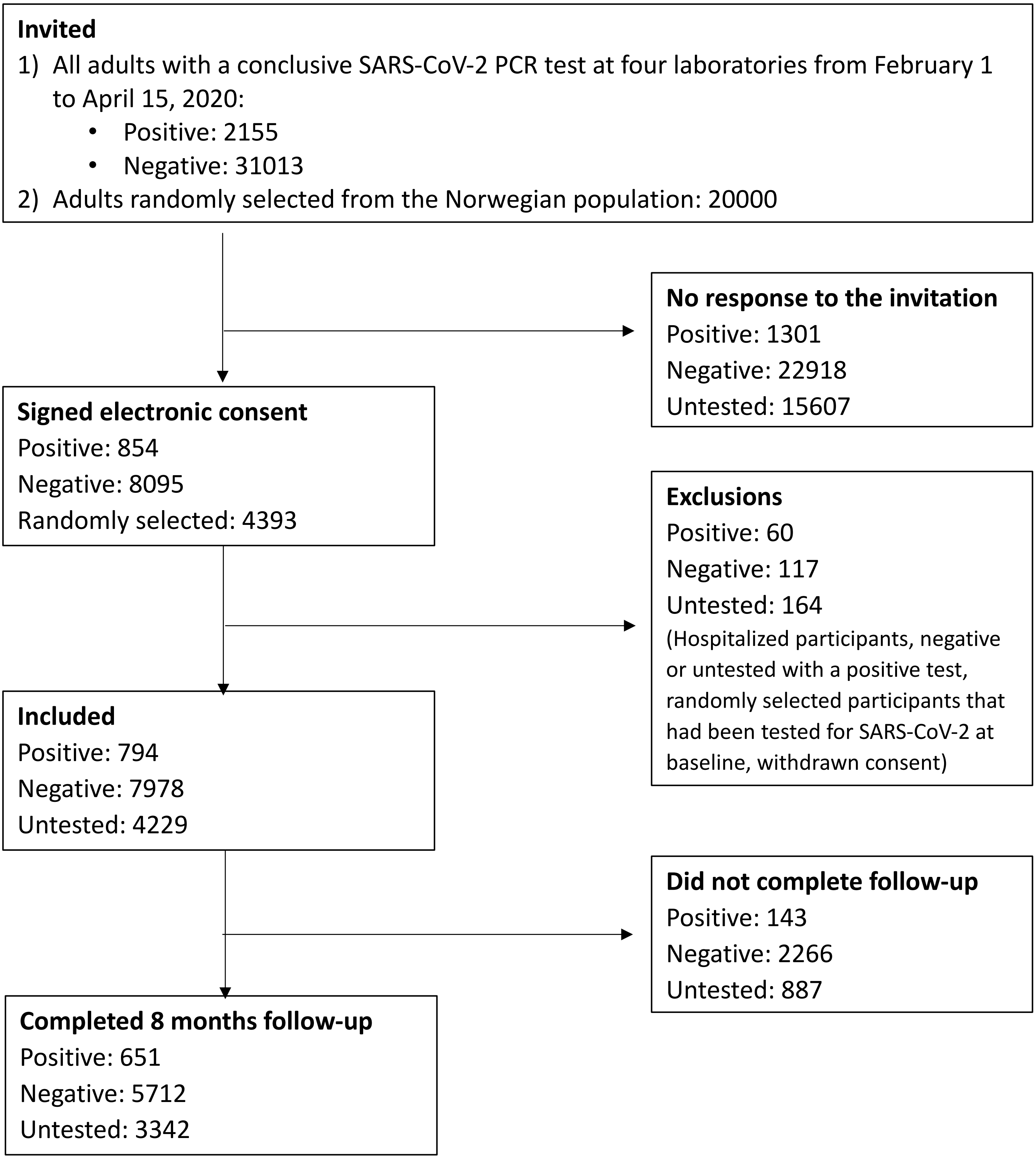

© 2021 Søraas A et al. *JAMA Network Open*.

Questionnaire 1

Certified translation of the baseline questionnaire (March-April 2020)
Only items relevant for the letter are presented.

Variables in red are presented in Table 1 of the letter

Check off every symptom that you have had over the course of the last three weeks:
Multiple answers are possible.

□ Fever
□ High fever (over 39)
□ Shortness of breath
□ Cough
□ Tiredness/exhaustion
□ Muscle pains Sore throat
□ Impaired sense of smell/taste
□ Blocked or runny nose
□ Headache
□ Stomach pains/nausea/diarrhoea
□ Other symptoms
□ No symptoms

© 2021 Søraas A et al. *JAMA Network Open*.

Questionnaire 2

Translation of the follow-up questionnaire (November 2020 and onwards)

Only items relevant for the letter are presented

Check off every symptom that you have had over the course of the last three weeks:
Multiple answers are possible.

□ Fever
□ High fever (over 39)
□ Shortness of breath
□ Cough
□ Tiredness/exhaustion
□ Muscle pains Sore throat
□ Impaired sense of smell/taste
□ Blocked or runny nose
□ Headache
□ Stomach pains/nausea/diarrhoea
□ Forgetfulness (direct translation of the Norwegian text: "Memory problems")
□ Confusion/Lack of concentration
□ Other symptoms
□ No symptoms

RAND 12 questionnaire included in follow-up

Items included in multivariable analysis as possible confounders for memory problems are bold. The official Norwegian translation was used in the study.

1. In general, would you say your health is:
   1 - Excellent
   2 - Very good
   3 - Good
   4 - Fair
   5 - Poor

© 2021 Søraas A et al. *JAMA Network Open*.

2. Has your health limited you in each of the following activities?

   a. The kinds or amounts of moderate activities you can do, like moving a table, vacuum cleaning, walking or gardening
   1 – Yes, limited a lot
   2 – Yes, limited a bit
   3 – No, not limited at all

   b. Climbing a few flights of stairs
   1 – Yes, limited a lot
   2 – Yes, limited a bit
   3 – No, not limited at all

3. **During the past four weeks, have you been unable to do certain kinds or amounts of work or housework because of your physical health?**
   1 – Yes
   2 – No

4. During the past four weeks, have you been unable to do certain kinds or amounts of work or housework because of your mental health?
   1 – Yes
   2 – No

5. **During the past 4 weeks, how much did pain interfere with your normal work (including both work outside the home and housework)?***
   1 - None
   2 - Very mild
   3 - Mild
   4 - Moderate
   5 - Severe
   6 - Very Severe

   **In Table 1 "Mild" to "Very Severe" pain were grouped together and the variable was dichotomized.*

© 2021 Søraas A et al. *JAMA Network Open*.

6. During the past four weeks, how much of the time have you felt calm and peaceful?

1 – All of the time
2 – Most of the time
3 – A good bit of the time
4 – Some of the time
5 – A little of the time
6 – None of the time

7. How much of the time during the past 4 weeks: Did you have a lot of energy?*

1 – All of the time
2 – Most of the time
3 – A good bit of the time
4 – Some of the time
5 – A little of the time
6 – None of the time

**In Table 1, "All of the time" to "A good bit of the time" pain were grouped together, and the variable was dichotomized.*

8. How much of the time during the past 4 weeks: Have you felt downhearted and blue?*

1 – All of the time
2 – Most of the time
3 – A good bit of the time
4 – Some of the time
5 – A little of the time
6 – None of the time

**In Table 1, "All of the time" to "A some of the time" pain were grouped together.*

9. During the past four weeks, has your health limited your social activities (like visiting with friends or close relatives)?

1 – All of the time
2 – Most of the time
3 – A good bit of the time
4 – Some of the time
5 – A little of the time
6 – None of the time

© 2021 Søraas A et al. *JAMA Network Open*.

The health transition item from the RAND 36 questionnaire was also included in follow-up and the results are reported in Table 1:

1. **Compared to one year ago, how do you rate your general health right now?**
    a. A lot better than one year ago
    b. A bit better than one year ago
    c. The same as one year ago
    d. A bit worse than one year ago
    e. A lot worse than one year ago

    **In Table 1 "A bit worse" and "A lot worse" pain were grouped together and the variable was dichotomized.*

© 2021 Søraas A et al. *JAMA Network Open*.
